# The Roles of Growth, Maturation, Physical Fitness, and Technical Skills on Selection for a Portuguese Under-14 Years Basketball Team

**DOI:** 10.3390/sports7030061

**Published:** 2019-03-08

**Authors:** Eduardo Guimarães, Adam Baxter-Jones, José Maia, Pedro Fonseca, Américo Santos, Eduardo Santos, Fernando Tavares, Manuel António Janeira

**Affiliations:** 1 CIFI2D, Faculty of Sports, University of Porto, 4200-450 Porto, Portugal; jmaia@fade.up.pt (J.M.); americosantos@fade.up.pt (A.S.); esantos@fade.up.pt (E.S.); ftavares@fade.up.pt (F.T.); janeira@fade.up.pt (M.A.J.); 2 College of Kinesiology, University of Saskatchewan, Saskatoon SK S7N 5B2, Saskatchewan, Canada; baxter.jones@usask.ca; 3 LABIOMEP, Porto Biomechanics Laboratory, 4200-450 Porto, Portugal; pedro.labiomep@fade.up.pt

**Keywords:** physical fitness, technical skills, biological maturation, training experience, youth basketball

## Abstract

This study investigated the roles of growth, maturation, physical fitness, and technical skills on selection onto an under-14 years basketball team. The sample consisted of 150 male players, aged 13.3 ± 0.7 years, divided into selected (SE—top players chosen by coaching staff to form an elite regional team) and non-selected (NSE—remaining players) groups. Anthropometry, body composition, biological maturation, and training experience data were collected using standard procedures. Physical fitness was assessed using the Yo-Yo IE2, sit-ups, handgrip, squat jump, countermovement jump, 3 kg medicine ball throw, 20 m sprint, and T-Test. Technical skills were acquired using the American Alliance for Health, Physical Education, Recreation, and Dance (AAHPERD)’s basketball-specific test battery. Groups were compared using a Student’s *t* test and multivariate analysis of covariance (MANCOVA), with training experience and biological maturation as covariates. A forward stepwise discriminant function analysis was employed to identify variables that maximized the separation between groups. The results showed that SE players were taller, had greater fat-free mass, greater strength, power, and agility, and were technically more skillful compared with NSE players (p < 0.05) when controlling for training experience and maturation. It was also found that players were best discriminated by the 3 kg medicine ball throw and control dribble, revealing the importance of qualified training to achieve excellence in youth basketball. 92.7% of the basketballers were correctly classified into their original groups. It is therefore confirmed that the additional effects of training experience and biological maturation positively influenced the performance of young basketball players. We recommend that coaches focus not only on players’ body sizes, but also on their skill level, especially during adolescence, when selecting teams in order to promote sustainable long-term development.

## 1. Introduction

Basketball is broadly characterized as a physiologically intermittent type of sport [1,2,3], requiring high levels of power, agility, and speed [4,5,6], all of which are closely linked to a player’s technical skills (TS) [7]. Furthermore, it is one of the most played team sports worldwide [8]. The game’s popularity is due to players´ athleticism, expressed by an optimal combination of body size, physique, motor abilities, and technical skills [9]. These features are often represented as multivariate profiles associated with success in response to training and competition, not only in senior but also in youth players [10].

The importance of basketball players’ body sizes, specifically tall stature, is well documented [6,9]. Although studies were not always able to differentiate young players from finalists, semi-finalists, and lower-ranked teams [11], most found these attributes to be highly associated with players’ competitive levels [12] and game performance [13]. Additionally, TS have been highlighted as important considerations to excel in basketball. For example, Brandão et al. [14] found that TS (passing, dribbling, and shooting) were strongly related with a team’s final standing, while te Wierike et al. [15] recommended that TS should be used by youth basketball coaches when selecting players and choosing the most appropriate specific position for them, since anthropometric and physiological differences tend to disappear as they grow [16]. These results were in line with the study by Karalejić et al. [17], who reported that correlations between anthropometric indicators and TS in youth athletes were relatively low, ranging from 0.23 to 0.54.

It is expected that differences in young players´ biological (sexual and skeletal) maturation may influence the magnitude of the above relationships. In fact, according to previous reports, maturity status acts as a discriminator of youth basketballers [12] as well as a predictor of game performance [13]. At the same time, results reported by te Wierike et al. [15] and Arede et al. [18] showed differences between players in physical fitness (PF) and TS sets which were related to maturity status, demonstrating that biological maturation also has a large impact on selection procedures. However, this is not always the case. In a study of Portuguese adolescent basketballers, PF and TS were independent of variations in maturation [19].

Available research considering the influence of biological maturation and training experience on young basketball players’ body sizes and performance is also scarce. For example, te Wierike et al. [15] controlled for differences in chronological age and age at peak height velocity (PHV), while Ramos et al. [11] controlled for differences in practice experience. However, to the best of our knowledge, research considering the simultaneous effects of biological maturation and training experience on players’ anthropometry, body composition, PF, and TS is apparently non-existent, as is research on their combined effects on selection onto teams. Nevertheless, since no single variable fully explains the multiple facets of performance, researchers systematically rely on multivariate approaches, including anthropometric (e.g., height, sitting height, weight, arm span, hand and leg length), body composition measures (e.g., fat and fat-free mass), and basketball-specific testing batteries to mark the ability to shoot, pass, dribble, and control the ball, as well as the capacity to move defensively [11,12,17,19,20]. Furthermore, time–motion analyses have shown that, during a basketball game, players engage in high-intensity and highly specific dynamic movements that change every 2 or 3 seconds [1,3,6]. Hence, PF is also commonly used to index players’ endurance, strength, jumping, velocity, and agility [18,21,22,23,24]. Moreover, when trying to uncover players’ multivariate profiles, the usual approach identified in the above-mentioned studies is to compare youths’ performances separated by: Competitive level [12,21,22,23], chronological age [17], maturity status [18,19,20,24], team competition standings [11,13], and specific positions [25,26].

Given that some of the available results are apparently inconsistent, there still exists a strong call to better nurture youth basketball players, especially during the pubertal period, when maturity status can impact a players’ growth, development, and performance [16]. The importance of this period for youth development is unquestionable, as systematic differences in maturity statuses among individuals of the same chronological age are well known [16]. In fact, along with the concerns of many researchers [13,15,27,28], the International Basketball Federation-World Association of Basketball Coaches (FIBA-WABC) [29] highlights that coaches working with 13–14 year old youngsters must expect that at this age some players appear to be physically bigger while passing through a stage of great emotional vulnerability. Therefore, coaches must understand that some players improve faster than others and must try to adapt to this [29].

Thus, using an extended set of variables that allows a unique multivariate approach as well as considering the simultaneous influence of two major youth performance confounders, the purposes of the present study were twofold: (1) To investigate the effects of training experience and biological maturation on body size and composition, PF, and TS of selected versus non-selected young basketball players, and (2) to identify the minimum set of predictors that best discriminate these two classes of players. With this in mind, we also intend to provide important and useful information that may help coaches to improve the development and selection of young players, as well as to increase success opportunities in their training sessions and competitions.

## 2. Materials and Methods

### 2.1. Participants

The study sample is part of a research project entitled *In search of excellence in sport—a mixed-longitudinal study in young athletes (INEX)*, conducted in the Porto metropolitan area in northern Portugal. In brief, this project aims to develop multivariate profiles of young male basketball, handball, soccer, volleyball, and water polo players, to model developmental trajectories both within and between individuals over time, to investigate predictors of success and the impact of the demands of the competition with increasing age.

In the present paper, we report cross-sectional data from 150 male basketball players aged 13.3 ± 0.7 years. Of these, sixteen were selected (SE) for an under-14 years team. The players competed in the first division of a regional basketball league and were chosen as the best ones from a total pool of 513 players by the Porto Basketball Association head and assistant coaches to integrate the under-14 regional team that competed in the 2016 Portuguese Inter-Associations National Championship. The players’ training experiences were 5.9 ± 2.4 years, and they trained 6–7 h·w^−1^. The non-selected players (NSE; n = 134) participated in the same first division and were randomly chosen from the same pool as a contrast to the SE ones. Their training experiences were 4.1 ± 2.4 years, and they trained 6–7 h·w^−1^. The Porto Basketball Association approved this project, and a written informed consent was obtained from parents or legal guardians of each player, and their individual assent was also obtained. This study was approved by the local Institutional Research Ethics Committee (CEFADE 13.2017).

### 2.2. Anthropometry and Body Composition

Height (cm), without shoes, was measured with the head positioned to the Frankfurt plane, using a stadiometer (Holtain Ltd., Crymych, UK) with a precision of 0.1 cm. Sitting height was measured while seated on a Harpenden (Holtain Ltd., Crymych, UK) sitting height table with legs hanging freely and arms resting on the thighs. Body mass was measured with a bio-impedance scale (Tanita® BC-418MA, Tanita Corp., Tokyo, Japan) with a precision of 100 g, and body fat and fat-free mass were derived according to the manufacturer’s formula. Arm span was measured with a segmometer (Rosscraft Innovations Inc., Vancouver, Canada). Hand length and hand breadth were measured with a sliding caliper (Holtain Ltd., Crymych, UK) with a precision of 0.1 cm. All measurements were taken according to the International Working Group on Kinanthropometry protocols [30] by experienced anthropometrists.

### 2.3. Biological Maturation

Biological maturation was assessed by predicting age from attainment of PHV. PHV is a somatic maturational milestone occurring during adolescences at approximately 14 years of age in boys. To predict when PHV would, or had, occurred, a maturity prediction equation using anthropometric measures was utilized [31]. A sex-specific formula based on age, sex, height, sitting height, and weight was used. The age from PHV occurrence estimates how many years the subject is from his age-at-PHV, and is termed “maturity offset”. A positive (+) maturity offset represents the number of years the participant is beyond PHV, whereas a negative (–) value represents the number of years before PHV.

### 2.4. Physical Fitness

PF was assessed as follows:

1. Aerobic fitness was measured with the Yo-Yo Intermittent Endurance Test—Level 2 (Yo-Yo IE2). All players performed repeated 40 m (2 x 20 m) runs with a 5 s active recovery period in between. The test ended when the participants failed twice to reach the finish line. The time and the total distance covered (m) was used as the test result [32];
2. Abdominal muscular strength and endurance was measured by recording the maximum number of sit-ups during 60 s. Each player performed two trials and the best trial was used as the test result [33];
3. Static strength was measured using a hand-held dynamometer (Takei Digital Grip Strength Dynamometer Model T.K.K.5401, Takei Scientific Instruments Co., Ltd., Tokyo, Japan), with players in a standing position. All players performed maximal handgrip strength (kg) on both hands, squeezing the dynamometer with maximal force and maintaining it away from the body with the arm extended. The mean of two best trials of each hand was used as the test result [34];
4. Lower body explosive power was measured with two vertical jumps (squat jump and countermovement jump), as advocated by Bosco et al. [35], using an AMTI OR6-WP force platform (Advanced Mechanical Technology Inc., Watertown, MA, USA) operating at 2000 Hz. Furthermore, jumping height (cm) was estimated [36]. Each player performed three trials for each vertical jump and the best one was used as the test result;
5. Upper body explosive power was measured with a 3 kg medicine ball throw (3 kg seated medicine ball throw). All players had to throw the ball straight forward as far as possible while seated on the floor with legs fully stretched and back against the wall. The distance (m) from the wall to where the ball landed was recorded and the mean of three trials was used as the test result [37];
6. Speed was measured with a 20 m sprint test, in which players ran at full speed. Each player performed two trials and the best one was used as the test result [33]. Time (s) was recorded using the photoelectric cells system Speed Trap II (Brower Timing Systems LLC., Draper, UT, USA);
7. Agility and body control were measured with the T-Test, in which players had to run and change directions rapidly in a T-shape pattern while maintaining balance and without loss of speed. Each player completed two trials and the best one was used as the test result [38]. Time (s) was obtained using the photoelectric cells system Speed Trap II (Brower Timing Systems LLC., Draper, UT, USA).

### 2.5. Technical Skills

Basketball-specific TS were assessed with the American Alliance for Health, Physical Education, Recreation and Dance (AAHPERD) [39] test battery:

1. Speed shot shooting: Players had to shoot the ball, get their own rebound, and dribble to another designated position, and repeat this sequence as quickly as possible during 60 s. Each successful shot counted as two points, while each unsuccessful one that hit the rim from above counted as one point;
2. Passing: Players performed chest passes against a wall marked with six specific targets of 60 × 60 cm and retrieved the ball while moving laterally during 30 s. Each pass hitting the target or the boundary counted as two points, while those hitting the intervening spaces on the wall counted as one point;
3. Control dribble: Players handled the ball while running as quickly as possible in a course defined by six cones, within a rectangle measuring 5.8 × 3.6 m;
4. Defensive movement: While keeping the basic defensive position, players performed lateral slides as quickly as possible without crossing their feet in a sequence of seven changes of direction.

Each player performed three trials for each test. The first one was a practice trial and the sum of the second and third trials was retained for further analysis. 

### 2.6. Data Quality Control

Data quality control consisted of a series of steps. First, all measurements were performed by trained personnel from the Kinanthropometry laboratory of the Faculty of Sport, University of Porto. Second, an in-field reliability study was performed so that a random sample of three-to-five subjects was re-measured every day. Third, reliability estimates were computed. The technical error of measurement (TEM) was 0.2 cm for height, 0.1 cm for sitting height, arm span, hand length, and hand breadth, 0.1 kg for weight, and 0.3 kg for both body fat and fat-free mass. ANOVA-based intraclass correlations (R) values for PF tests ranged from 0.82 (sit-ups) to 0.99 (3 kg seated medicine ball throw), and from 0.83 (speed shot shooting) to 0.96 (defensive movement) in TS.

### 2.7. Statistical Analysis

Results are presented as means and standard deviations (M ± sd). Normality and homogeneity of variances were checked, and no violations were found. Groups were compared in a univariate fashion using a Student’s *t*-test. Cohen’s *d* were also calculated and interpreted as follows: <0.20 (trivial), 0.20 to 0.59 (small), 0.60 to 1.19 (moderate), 1.20 to 1.99 (large), 2.00 to 3.90 (very large), and >4.00 (extremely large) [40]. The effects of training experience and biological maturation (maturity offset) on anthropometry, body composition, PF, and TS were examined using a multivariate analysis of covariance (MANCOVA), and eta squared (η^2^) was used as a measure of explained variance. Further, using only variables that had a statistically significant effect, a forward stepwise discriminant function analysis was employed to identify the smallest set of variables that maximizes the differences between the groups. Finally, results from the confusion matrix showed how precise the smallest variable set obtained from the discriminant function was in recovering the original grouping of all subjects. All data analyses were done using SPSS 23.0 (IBM Corp., Armonk, New York, US), and the significance level was set at 5%.

## 3. Results

Descriptive statistics for age, training experience, anthropometric and body composition, biological maturation, PF, and TS components are shown in Table 1. In general, significant differences were found between groups in favor of those selected (*p* < 0.05) apart from arm span, body fat, sit-ups, countermovement jumps, and squat jumps (*p* > 0.05).

When controlling for training experience and biological maturation, the MANCOVA results (Table 2) for each of the three sets of variables—anthropometry/body composition, PF, and TS—showed significant results in all multivariate tests, and the η^2^ varied from 9% (TS) to 27% (PF). In the anthropometry/body composition set, only two variables significantly favored the SE group—height and fat-free mass. In the PF set, SE players outperformed NSE players in handgrip strength, T-Test, and 3 kg seated medicine ball throw. Additionally, SE players were more skillful in all tests, but on defensive movement, no significant differences were found.

Table 3 reports the main results of the forward stepwise discriminant function, and shows the best smaller set of the previous eight variables that best discriminates the groups and are in order of importance (3 kg seated medicine ball throw and control dribble).

**Figure 1:**
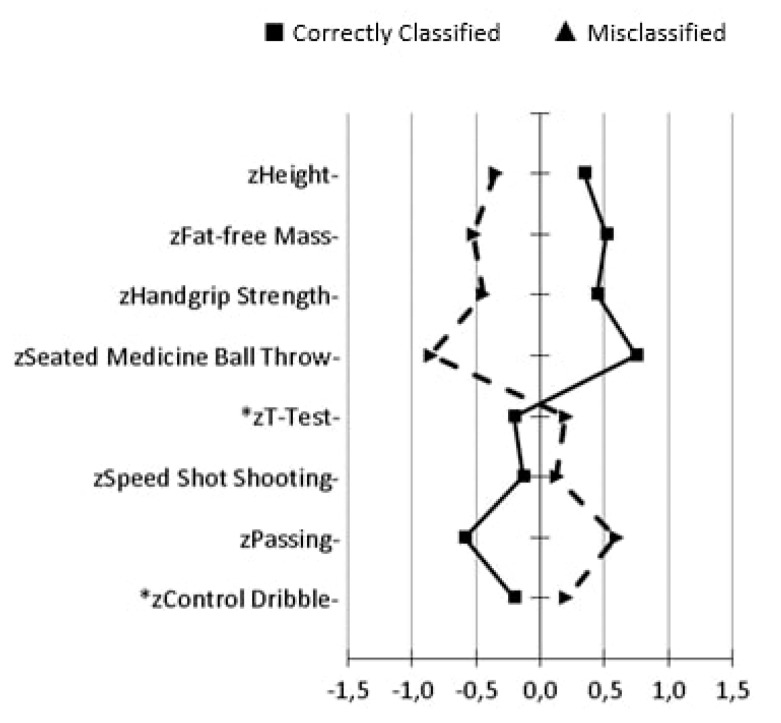
Multivariate graphical representation of correctly and misclassified selected players (all variables were standardized). (*) = less time is better performance.

From the confusion matrix results (Table 4), eight SE players were correctly classified in their original group, while in the NSE ones only three were misclassified. The players were correctly classified as SE and NSE in 92.7% of cases. Figure 1 displays the multivariate graphical profiles of correctly and misclassified SE players. Misclassified cases were smaller, had lower fat-free mass, and performed worst in handgrip strength, 3 kg seated medicine ball throw, T-Test, and control dribble tests than correctly classified, with the exception of both speed shot shooting and passing tests.

## 4. Discussion

This study investigated the multivariate profiles of groups of selected (SE) versus non-selected (NSE) Portuguese young basketball players, controlling for training experience and biological maturation. It was found that a 3 kg seated medicine ball throw and a control dribble were the variables that separated these groups of young players.

### 4.1. Anthropometry and Body Composition

Consistent with previous research in young basketball players [12,13,25], our study showed that SE players were taller and heavier, with greater hand length and hand breadth, and higher values in fat-free mass. Further, they had more years of training experience and were more advanced in biological maturation, characteristics commonly observed in other studies contrasting players from different competitive [11] and maturational levels [18,28]. It is possible that these two factors explain a major part of the physical differences observed between SE and NSE players. However, when controlled for training experience and biological maturation, significant differences in height and fat-free mass still remained. Usually, differences in height tend to disappear when the effect of biological maturation is accounted for [41,42,43,44,45]. However, in youth basketballers, tall stature is often the first marker for selection [17,28]. Taken together, these results suggest that coaches should be careful when selecting players only based on anthropometric attributes, as they may simply be related to an advanced maturity status [15,18,24,28]. Differences in fat-free mass also remained, probably because of the training quality of SE players’ teams (they play in highly competitive teams), and the selection process may also have played a role.

### 4.2. Physical Fitness 

Consistent with documented research in young basketball players [11,12,13,21,25], our study also showed that SE players, i.e., top-level young basketball players, had higher aerobic fitness, static strength, upper body explosive power, and were faster and more agile. However, when controlling for training experience and biological maturation, significant differences only remained in static handgrip strength, upper body explosive power, and agility. Unfortunately, we were not able to find any other studies with young basketball players where our two major predictors were used as covariates to control for players’ performance. However, Ramos et al. [11], after adjusting only for training experience, also found that the best players were more agile and had greater upper body explosive power. Moreover, this trend is in line with other findings in soccer [41,42,43] and handball [44,45] that used the same two covariates as employed in this study. Taken together, the present results suggest that high-level players’ physical responses to training and competition seem to be in accordance with the specificities of the game. Since they performed better in these three physical abilities, it is expected that they may be more prepared to face some of the game challenges, namely, rapid changes of direction and even to shoot, pass, or dribble the ball with greater effectiveness.

### 4.3. Technical Skills

As expected, SE players performed better in all specific TS. This evidence is partially consistent with previous reports in young basketballers, as offensive TS (speed shot shooting, passing, and control dribble) have been previously shown to be strongly related with the final team classification [14,46]. In fact, the high importance of TS to differentiate top- and low-level players is also evident in other studies with young soccer players [42] and young field hockey players [47]. However, when controlling for training experience and maturation, significant differences only remained in speed shot shooting, passing, and control dribble, suggesting that their training regimens may concentrate on the offensive dimension of the game, i.e., are highly focused on players’ ability to shoot, pass, and dribble. Interestingly, this novel trend is also confirmed by studies using game-related statistics as technical performance predictors. For example, Torres-Unda et al. [12] reported that elite players score more points per game, i.e., they are determinant for teams’ offensive success. Even so, it is also possible that coaches may play an important role in these results, because at this age category they favor/select highly skilled players [17]. 

### 4.4. Discriminant Function

The variables that best discriminated SE players from NSE players were the 3 kg seated medicine ball throw and control dribble. This means that upper body explosive strength (linked itself to shooting, passing, and dribbling the ball) and the capacity to handle ball control while running as fast as possible (decisive to maintain the ball possession, change direction, and progress in court) were the variables that maximized the differences between these two groups. These results are in part consistent with previous reports using discriminant function analysis in youth basketball. Indeed, Ramos et al. [11] found that speed discriminated finalist players from lower-ranked players (in youth male teams), while a combination of speed and upper body explosive strength discriminated semifinalists from lower-ranked players (in youth female teams). A similar trend is also present in studies in youth soccer and handball players. For example, Vaeyens et al. [42] showed that among under-13 and under-14 soccer players, speed (shuttle sprint and 30 m dash) and soccer-specific technical skills best discriminated players with distinct skill levels (elite, sub-elite, and non-elite), while among under-15 and under-16 players, endurance (shuttle run), skills, and speed were the best discriminators. Furthermore, Mohamed et al. [44] revealed that height, speed, and agility were the most important variables to discriminate young elite and non-elite handball players. Consistently, Matthys et al.’s [45] data showed that speed and agility were the indicators that best separated under- 14 elite and non-elite handball players, while agility, strength, endurance, and power and flexibility were the strongest discriminating factors among under-16 and under-18 players, respectively. Taken together, our findings in youth basketball are similar to that of youth soccer and handball, emphasizing the importance of TS and functional variables as compared to anthropometric and body composition information on youth selection and development. Furthermore, these discriminant markers are in line with game-specific requirements, and also match with suggested traits essential to success [11,15,17,18]. Despite the efficiency of the discriminant classification (92.7% of the cases were correctly classified in their original groups), eight SE players were misclassified. Interestingly, these misclassified players performed better in speed shot shooting and passing, while the correctly classified ones were taller, had higher fat-free mass, and performed better in handgrip strength, 3 kg seated medicine ball throw, T-Test, and control dribble. This may point out that coaches responsible for selection at this age category not only favor players with higher values in body size and physical fitness (corrected classified), but also players with a high degree of technical expertise (misclassified) in order to set up a team capable to positively respond to all game challenges and, consequently, with greater potential to win a national championship. Indeed, the selection process represented in the current study demonstrate that coaches took into account players who are apparently fitter for physical contact near the basket as well as others who are more skillful at playing outside the perimeter. 

This study is not without limitations. First, sample sizes across groups were not homogeneous. However, such assumptions are unrealistic when considering players specifically selected by coaches or technical staff to represent and compete for a regional or national team, as well as players from different competitive levels. This is a consistent finding across the literature [11,12,45]. Second, our sample is from the Porto Basketball Association from the Porto district, located in the north of Portugal. As such, a careful generalization of our results is required. Nevertheless, there are also some strong points that need to be highlighted. First, the sample comprises players from different levels of sporting excellence. Second, the extended set of variables used allowed a unique multivariate approach oftentimes lacking in studies of this nature [11,13,17]. Third, by controlling not only for biological maturation [15] but also for training experience, we were able to better understand the role of these two confounders on players’ selection procedures.

## 5. Conclusions

In summary, the results of our study confirm that the additional effects of training experience and biological maturation positively influence the performance of young basketball players. Furthermore, even when controlled for, SE players were taller and more muscular, had greater strength, power, and agility, and also were more technically skillful. Additionally, a follow-up discriminant analysis showed that SE and NSE players differ greatly in PF and TS, revealing the importance of qualified training to achieve excellence in youth basketball. Despite these results, it seems necessary to carry out longitudinal studies using a multi-dimensional approach including other components (e.g., tactical skills, perceptual–cognitive skills, individual psychological characteristics, training and competition information, and contextual factors) for a better understanding of the complex path to success. Finally, it is important for coaches, trainers, and experts in selection to be more aware of the tremendous influence of training experience and biological maturation when recruiting and selecting young basketball athletes. We also recommend coaches to focus not only on players’ body sizes, but also on their skill levels, especially during adolescence, in order to promote sustainable long-term development. This seems to be crucial to increase the opportunities of young players to succeed in their training demands as well as in competition expectations.

## Figures and Tables

**Table 1:** Descriptive statistics (M ± sd) for age, training experience, anthropometric and body composition, biological maturation, physical fitness, and technical skills variables of selected and non-selected young basketball players.

Variable	Selected (n = 16)	Non-Selected (n = 134)	Mean Difference (95% CI)	Cohen’s *d*
	M ± sd	M ± sd		
Age (years)	13.6 ± 0.4	13.2 ± 0.7	0.4 (0.0, 0.7) *	0.70
Training Experience (years)	5.9 ± 2.4	4.1 ± 2.4	1.9 (0.6, 3.1) **	0.75
Anthropometry and Body Composition				
Height (cm)	177.4 ± 6.2	163.1 ± 10.0	14.3 (9.2, 19.3) **	1.72
Weight (kg)	66.1 ± 8.2	52.8 ± 10.7	13.3 (7.8, 18.8) **	1.40
Arm Span (cm)	174.7 ± 20.9	167.5 ± 14.8	7.3 (–0.8, 154)	0.40
Hand Length (cm)	18.6 ± 0.8	17.3 ± 1.3	1.3 (0.6, 1.9) **	1.20
Hand Breadth (cm)	21.7 ± 0.9	19.7 ± 1.6	2.0 (1.2, 2.8) **	1.54
Body Fat (kg)	11.1 ± 3.2	10.0 ± 3.5	1.1 (–0.7, 3.0)	0.33
Fat-free Mass (kg)	55.0 ± 5.8	42.8 ± 8.3	12.2 (8.0, 16.4) **	1.70
*Biological Maturation*				
Maturity Offset (years)	0.9 ± 0.6	–0.4 ± 0.9	1.3 (0.8, 1.8) **	1.70
Physical Fitness				
Yo-Yo IE2 (m)	1097.5 ± 411.6	714.6 ± 322.7	382.9 (209.0, 556.8) **	1.04
Sit-Ups (repetitions)	34.5 ± 6.8	34.5 ± 7.4	–0.1 (–4.0, 3.9)	0.00
Handgrip Strength (kg)	33.6 ± 5.5	25.1 ± 6.3	8.5 (5.2, 11.7) **	1.44
Squat Jump (cm)	26.3 ± 5.1	25.1 ± 6.2	1.2 (–2.4, 4.7)	0.21
Countermovement Jump (cm)	29.3 ± 7.2	30.0 ± 5.8	–0.7 (–4.2, 2.7)	–0.11
3 kg Seated Medicine Ball Throw (m)	5.0 ± 0.6	3.5 ± 0.7	1.5 (1.1, 1.8) **	2.30
20 m Sprint (s)	3.3 ± 0.2	3.7 ± 0.3	–0.3 (–0.5, –0.2) **	–1.57
T-Test (s)	9.2 ± 0.4	10.0 ± 0.6	–0.8 (–1.0, –0.6) **	–1.57
Technical Skills				
Speed Shot Shooting (points)	36.3 ± 3.9	30.8 ± 5.8	5.5 (2.5, 8.4) **	1.11
Passing (points)	98.6 ± 11.3	84.1 ± 12.9	14.5 (7.8, 21.2) **	1.20
Control Dribble (s)	15.4 ± 0.7	17.3 ± 1.5	–1.9 (–2.6, –1.2) **	–1.62
Defensive Movement (s)	18.5 ± 1.3	20.4 ± 2.2	–1.9 (–3.0, –0.8) **	–1.05

CI = confidence intervals; (*) = *p* < 0.05; (**) = *p* < 0.01.

**Table 2:** Multivariate analyses of covariance (MANCOVA) with training experience and maturity offset as covariates.

Variable	Selected (n = 16)	Non-Selected (n = 134)	Multivariate Test		Univariate Test	
	AdjM ± SE	AdjM ± SE	F	η^2^	F	η^2^
Anthropometry and Body Composition			3.65 **	0.15		
Height (cm)	166.8 ± 1.1	164.4 ± 0.3			4.11 *	0.03
Weight (kg)	57.2 ± 1.7	53.8 ± 0.5			3.19	0.02
Arm Span (cm)	163.8 ± 3.1	168.8 ± 1.0			2.29	0.02
Hand Length (cm)	17.5 ± 0.2	17.4 ± 0.1			0.01	0.00
Hand Breadth (cm)	20.4 ± 0.3	19.8 ± 0.1			2.81	0.02
Body Fat (kg)	10.4 ± 0.9	10.1 ± 0.3			0.09	0.00
Fat-free Mass (kg)	46.8 ± 1.0	43.8 ± 0.3			7.55 **	0.05
Physical Fitness			5.26 ***	0.27		
Yo-Yo IE2 (m)	832.1 ± 96.2	729.0 ± 29.0			1.01	0.01
Sit-Ups (repetitions)	32.8 ± 2.3	35.1 ± 0.7			0.82	0.01
Handgrip Strength (kg)	29.0 ± 1.3	25.6 ± 0.4			5.78 *	0.05
Squat Jump (cm)	25.8 ± 1.9	25.2 ± 0.6			0.09	0.00
Countermovement Jump (cm)	26.8 ± 1.8	30.2 ± 0.6			3.04	0.02
3 kg Seated Medicine Ball Throw (m)	4.3 ± 0.1	3.6 ± 0.0			24.15 **	0.16
20 m Sprint (s)	3.5 ± 0.1	3.7 ± 0.0			3.35	0.03
T-Test (s)	9.5 ± 0.2	10.0 ± 0.1			7.76 **	0.06
Technical Skills			3.60 **	0.09		
Speed Shot Shooting (points)	34.7 ± 1.5	31.0 ± 0.5			5.52 *	0.04
Passing (points)	93.6 ± 3.5	84.7 ± 1.1			5.82 *	0.04
Control Dribble (s)	15.8 ± 0.4	17.2 ± 0.1			11.80 **	0.08
Defensive Movement (s)	19.5 ± 0.6	20.2 ± 0.2			1.22	0.01

AdjM = adjusted mean; SE = standard error; (*) = *p* < 0.05; (**) = *p* < 0.01; (***) = *p* < 0.001.

**Table 3:** Summary of stepping summary in forward stepwise discriminant analysis.

Step	Entered	Wilks’s Lambda	Approx. F-Ratio	*p*-Value
1	3 kg Seated Medicine Ball Throw (m)	0.692	64.93	*p* < 0.01
2	Control Dribble (s)	0.665	36.45	*p* < 0.01

**Table 4:** Classification matrix of selected and non-selected young basketball players.

Group	Selected	Non-Selected	% Correct
Selected	8	8	50.0
Non-Selected	3	131	97.8
Total	11	137	92.7
